# Serum neurofilament indicates accelerated neurodegeneration and predicts mortality in late-stage Parkinson’s disease

**DOI:** 10.1038/s41531-023-00605-x

**Published:** 2024-01-09

**Authors:** Anika Frank, Jonas Bendig, Nils Schnalke, Lisa Klingelhoefer, Heinz Reichmann, Katja Akgün, Tjalf Ziemssen, Björn H. Falkenburger

**Affiliations:** 1 Department of Neurology, Faculty of Medicine and University Hospital Carl Gustav Carus, Technische Universität Dresden, Dresden, Germany; 2 German Center for Neurodegenerative Diseases (DZNE), Dresden, Germany; 3 Center of Clinical Neuroscience, Department of Neurology, Technische Universität Dresden, Dresden, Germany

**Keywords:** Prognostic markers, Neurodegeneration

## Abstract

Different stages of Parkinson’s disease (PD) are defined by clinical criteria, while late-stage PD is marked by the onset of morbidity milestones and rapid clinical deterioration. Based on neuropathological evidence, degeneration in the dopaminergic system occurs primarily in the early stage of PD, raising the question of what drives disease progression in late-stage PD. This study aimed to investigate whether late-stage PD is associated with increased neurodegeneration dynamics rather than functional decompensation using the blood-based biomarker serum neurofilament light chain (sNfL) as a proxy for the rate of neurodegeneration. The study included 118 patients with PD in the transition and late-stage (minimum disease duration 5 years, mean (SD) disease duration 15 (±7) years). The presence of clinical milestones (hallucinations, dementia, recurrent falls, and admission to a nursing home) and mortality were determined based on chart review. We found that sNfL was higher in patients who presented with at least one clinical milestone and increased with a higher number of milestones (Spearman’s *ρ* = 0.66, *p* < 0.001). Above a cutoff value of 26.9 pg/ml, death was 13.6 times more likely during the follow-up period (95% CI: 3.53–52.3, *p* < 0.001), corresponding to a sensitivity of 85.0% and a specificity of 85.7% (AUC 0.91, 95% CI: 0.85–0.97). Similar values were obtained when using an age-adjusted cutoff percentile of 90% for sNfL. Our findings suggest that the rate of ongoing neurodegeneration is higher in advanced PD (as defined by the presence of morbidity milestones) than in earlier disease stages. A better understanding of the biological basis of stage-dependent neurodegeneration may facilitate the development of neuroprotective means.

## Introduction

Parkinson’s disease (PD) is a progressive neurological disorder that impacts millions of individuals globally. Although current treatments can provide symptomatic relief, there is no therapy available that can effectively slow down the progression of PD over time. Clinically, the course of PD is categorized into distinct stages, starting with the prodromal stage that precedes the formal diagnosis^1^. In clinical trials, early-stage PD is typically defined by the Hoehn and Yahr stages 1 and 2^2^, while advanced-stage PD is often characterized by the presence of motor fluctuations^3^. Late-stage PD is primarily identified by dopamine-resistant motor symptoms and non-motor symptoms^4^. However, the biological underpinnings of these stages remain largely unclear. Interestingly, degeneration in the dopaminergic system reaches its peak within the initial four years after diagnosis^5^, suggesting that the transitions between subsequent stages are not solely attributed to dopamine loss. PD pathology extends beyond the dopaminergic system, affecting other neurotransmitter systems^6–8^, and Lewy pathology progressively spreads throughout the nervous system^9,10^. Furthermore, functional decline in PD may arise from the weakening of compensatory mechanisms rather than direct neurodegeneration. For instance, maladaptive plasticity in the striatum is believed to contribute to the development of dyskinesias and motor fluctuations^11,12^.

Although the course of the disease varies considerably in the early stages and appears stable for a significant length of time, the late stage, in contrast, is characterized by a relatively rapid and uniform clinical deterioration^13^. Specifically, the occurrence of morbidity milestones, which include visual hallucinations, recurrent falls, the onset of dementia, and admission to a nursing facility, have been associated with poor prognosis heralding the final five years of the disease^13^. Even in patients with long-term deep brain stimulation (DBS), the same rapid progression was found after the onset of morbidity milestones—in spite of more benign disease trajectories in DBS patients in early to mid-stage disease^14^. These findings indicate a pathophysiological process, which is inherent to the last phase of the disease or an age-modulated exponential progression culminating in comparable trajectories. Such an accelerated progression or “neurodegenerative storm” in the terminal stage can be illustrated in the form of a hockey-stick effect, which closes a steady linear deterioration with a steep upward curve at the end^13,14^. The underlying factors for these processes are unknown, however, both models could be caused by a failing stress compensation in already critically degenerated areas where only minor cellular loss acts as the straw breaking the camel’s back. Another possible mechanism is the spread of alpha-synuclein beyond the midbrain structures, including the cortex and the limbic system, which results in a pronounced increase in affected brain volume.

Biomarkers such as serum neurofilament light chain (sNfL) could help to determine the underlying mechanism in this stage of the disease as sNfL reports neuro-axonal damage and is elevated in many neurodegenerative diseases including PD^15–19^. Although sNfL is not specific for PD induced neurodegeneration but rather reflects any neuro-axonal damage—peripheral or central—there is a solid body of evidence supporting its role as a marker for ongoing neurodegeneration^20^. For example, sNfL levels were already elevated during the asymptomatic phase in mutation carriers of familial Alzheimer’s disease and sNfL dynamics accelerated in individuals transitioning from the presymptomatic to the symptomatic stage^21^. In many other neurodegenerative disorders like amyotrophic lateral sclerosis, atypical parkinsonism and PD, higher levels of sNfL were shown to be associated with faster disease progression and higher brain atrophy rates^22^. Furthermore, in a number of neurological disorders, effective disease-modifying therapies were shown to normalize NfL levels linking NfL dynamics to the clinical efficacy of the treatment^23–25^. Based on this, sNfL can be regarded as a measure of the intensity of ongoing neurodegeneration^26^.

We hypothesize that the rapid increase in disability after the occurrence of a clinical milestone results not only from functional decompensation but from accelerated neurodegeneration. To test this hypothesis, we measured sNfL in a cohort of patients with PD at least 5 years after diagnosis and compared patients with and without clinical milestones. Furthermore, we investigated the predictive value of sNfL for all-cause mortality. In brief, we find increased sNfL levels in patients with morbidity milestones, suggesting a high rate of ongoing neurodegeneration in late-stage PD, which entails the potential to develop stage-specific therapeutics to prevent the rapid clinical deterioration heralded by the onset of morbidity milestones.

## Results

118 patients with PD (disease duration of at least 5 years) were included in the study; demographic and clinical data are summarized in Table 1. Pairwise comparisons between the groups in Table 1 are displayed in Supplementary Table 1. A large proportion of study participants were enrolled in an observational register study for patients with DBS. We have shown previously that there are no differences between sNfL values between patients treated with DBS and patients without DBS when correcting for age and disease duration in the same cohort^27^. As expected from the use of an advanced therapeutic option, patients treated with DBS had a longer disease duration (16.7 ± 6.5 vs 9.2 ± 5.2, *p* < 0.0001, Mann–Whitney *U* test), and it was more likely for the milestones dementia (*p* = 0.0007, Fisher’s exact test) or recurrent falls (*p* = 0.0418, Fisher’s exact test) to be present.

**Table 1:** Demographic and clinical data.

	Entire cohort (*n* = 118)	No milestone at baseline (*n* = 42)	≥1 Milestone at baseline (*n* = 76)	Alive at Last FU (*n* = 98)	Passed During FU (*n* = 20)
Age, years	66 (±8)	**63** (±**8)**	**68** (±**8)**	**65** (±**8)**	**72** (±**7)**
Age at PD onset, years	51 (±8)	52 (±9)	51 (±7)	51 (±8)	52 (±5)
Disease duration, years	15 (±7)	**10** (±**5)**	**17** (±**7)**	**14** (±**7)**	**20** (±**6)**
Sex, male/female, *n* (male,%)	84/34 (71.2)	29/13 (69.0)	55/21 (72.3)	70/28 (71.4)	14/6 (70.0)
Hoehn & Yahr stage	2 (2–3)	**2 (2–3)**	**3 (2–4)**	**2 (2–3)**	**4 (3–4)**
LEDD, mg	800 (±411)	730 (±288)	838 (±463)	799 (±423)	801 (±358)
UPDRS III score (ON medication)	23 (±12)	**20** (±**11)**	**25** (±**11)**	**21** (±**10)**	**36** (±**11)**
MoCA score	24 (±6)	**27** (±**2)**	**22** (±**6)**	**26** (±**3)**	**16** (±**8)**
Total number of milestones at baseline	1 (0–2)	**0 (0–0)**	**2 (1–3)**	**1 (0–2)**	**3 (2–4)**
sNfL, pg/ml	22.7 (±16.1)	**14.5** (±**5.6)**	**27.3** (±**18.1)**	**18.3** (±**8.3)**	**44.3** (±**25.5)**

The data are provided for the entire cohort as well as group comparisons for patients with and without a milestone at the study’s baseline, as well as patients who survived or passed away during the study follow-up. Statistical significance between these groups (no milestone vs. ≥ 1 milestone and alive vs. deceased) was assessed using the Mann–Whitney *U* test, and values below < 0.05 are indicated in bold. For categorical variables, the Chi-square test (*χ*²) was employed. Significance levels of pairwise comparisons between the groups are displayed in Supplementary Table 1. Data represent mean values ± standard deviation except for Hoehn & Yahr stage and total number of milestones (Median, Interquartile range) as well as sex (*n*/%). *FU* follow-up, *LEDD* levodopa equivalent daily dose, *MoCA* Montreal Cognitive Assessment, *sNfL* serum neurofilament light chain, *UPDRS III* Unified Parkinson’s Disease Rating Scale Part III.

### Serum NfL is associated with clinical milestones and discriminates between patients with or without clinical milestones

**Fig. 1:**
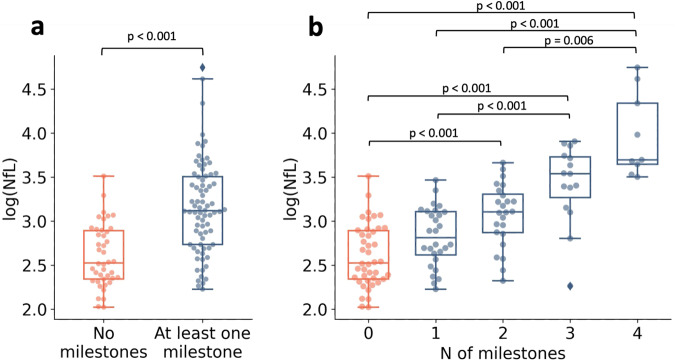
sNfL increases with the presence of clinical milestones. Comparison of log(NfL) values for patients with no milestone or at least one clinical milestone (**a**) and of log(NfL) values stratified by the number of clinical milestones (**b**). Data are depicted as boxplots (center line—median, box limits—upper and lower quartiles, whiskers—1.5× interquartile range, diamonds—outliers) overlaid with individual markers for each patient (dots). Only significant differences between groups are depicted in the figure. Groups were compared using Mann–Whitney *U* Test (**a**) or a posthoc Dunn-Test after a significant Kruskal–Wallis test (*p* < 0.001) adjusted for false discovery rate using the Benjamini–Hochberg method (**b**).

At baseline, 42 patients showed no clinical milestone, while 76 showed at least one milestone, confirming the advanced stage of our cohort. sNfL was significantly higher in patients with at least one clinical milestone than in patients without a clinical milestone (Fig. 1a), and sNfL correlated positively with the number of clinical milestones (Fig. 1b, Spearman’s *ρ* = 0.672, *p* < 0.001).

Linear models using log(NfL) as the dependent variable revealed that 51.1% of the variance in log(NfL) is explained by the combination of the four milestones (Adjusted *R*^2^ = 0.511, *p* < 0.001). The effects of all milestones on log(NfL) remained significant after adding age, disease duration and sex to the linear model and only age significantly improved the model parameters (Adjusted *R*^2^ = 0.581, *p* < 0.001). When using age-adjusted sNfL *Z* scores as the dependent variable, the milestones dementia, visual hallucinations and recurrent falls contributed significantly to the linear model, while admission to a nursing facility did not (adjusted *R*^2^ = 0.371, *p* = 0.008, *p* < 0.001, *p* = 0.025 and 0.132, respectively). The individual effects of each variable on log(NfL) and age-adjusted sNfL *Z* scores were further explored with univariate linear regression models (Supplementary Table 2).

### Serum NfL predicts death within three years

As mentioned above, clinical milestones typically occur about 5 years prior to death^13^. The mean follow-up period between the sNfL measurement and the last follow-up chart review was 28.4 ± 8.7 months. In our cohort of 118 patients with advanced PD, 20 patients died during that follow-up period. These patients showed significantly higher age, disease duration and more severe symptoms as reported by Hoehn & Yahr stage, UPDRS III and MoCA scores at the baseline visit (Supplementary Table 1). There were no significant differences in age of onset, sex distribution, or levodopa equivalent daily dose (LEDD).

Surprisingly, the presence of one clinical milestone alone at baseline did not predict death during follow-up in our cohort (*p* = 0.13, Table 2). However, the presence of at least two clinical milestones increased the risk of death during follow-up by a factor of 21.0 (Table 2), and more milestones were associated with a higher risk of death (HR = 3.86 per additional milestone, Table 2). Among individual milestones, dementia showed the highest hazard ratio (HR = 11.6, Table 2), while recurrent falls showed the lowest hazard ratio and was above the threshold for significance (*p* = 0.051, Table 2). Other clinical parameters like lower MoCA scores, higher UPDRS III scores and a higher Hoehn & Yahr stage were also significantly associated with a higher risk of death during follow-up (Table 2). Consistent with the hypothesis of accelerated neurodegeneration in the final phase of the disease, higher log(NfL) at baseline proved to be a strong risk factor for death during follow-up independent of age at baseline, disease duration and sex (HR = 12.9 per 1 point increase, Table 2).

**Table 2:** Hazard ratios associated with sNfL and other risk factors.

Characteristic	HR	95% CI	*p* value
log(NfL) (1 point increase)	13.9	4.93, 39.3	**<0.001**
sNfL > 26.9 pg/ml	13.6	3.53, 52.3	**<0.001**
≥1 milestone	5.04	0.62, 40.9	0.13
≥2 milestones	21.0	2.67, 166	**0.004**
Number of milestones (increase by 1)	3.86	2.22, 6.72	**<0.001**
Milestone dementia	11.6	2.55, 52.6	**0.002**
Milestone visual hallucinations	5.53	1.97, 15.6	**0.001**
Milestone nursing home admission	4.58	1.61, 13.0	**0.004**
Milestone recurrent falls	4.55	0.99, 20.9	0.051
MoCA (1 point decrease)	1.21	1.14, 1.29	**<0.001**
UPDRS III (1 point increase)	1.09	1.04, 1.14	**<0.001**
Hoehn & Yahr (1 point increase)	2.80	1.65, 4.74	**<0.001**

Hazard ratios were obtained from multiple Cox-regression models predicting death in the observation period adjusted for age at baseline, disease duration and sex. One point increase in log(NfL) corresponds to a 2.718-fold increase of sNfL. *CI* confidence interval, *HR* hazard ratio, *MoCA* Montreal Cognitive Assessment, *sNfL* serum neurofilament light chain, *UPDRS III* Unified Parkinson’s Disease Rating Scale Part III. *p* values of significant Hazard Ratios are marked in bold.

**Fig. 2:**
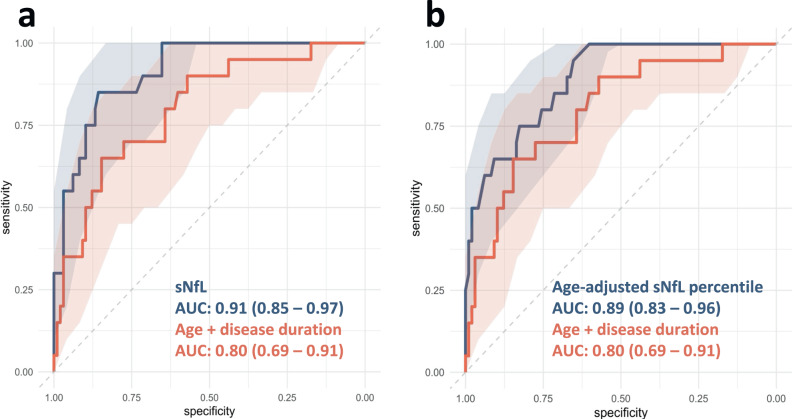
ROC curves for sNfL and age-adjusted sNfL percentiles. ROC curves for the prediction of death during follow-up by sNfL (**a**, blue curve) and age-adjusted sNfL percentiles (**b**, blue curve) at baseline. For reference, a ROC curve of a logistic regression model containing age and disease duration was added in both panels (**a**, **b**, orange curves). 95% confidence intervals (bootstrap method, 2000 stratified intervals) are depicted around each ROC curve. AUC area under the curve, sNfL serum neurofilament light chain.

To further test the predictive quality of sNfL for death during follow-up and to identify a cutoff value with high sensitivity and specificity, we fitted ROC curves (Fig. 2a). The area under the curve for the prediction of death within three years by (unadjusted) sNfL alone was 0.91 (0.85–0.97). The optimal cutoff value was sNfL = 26.9 pg/ml (determined using the Youden Index), resulting in a sensitivity of 85.0% and a specificity of 85.7%.

**Fig. 3:**
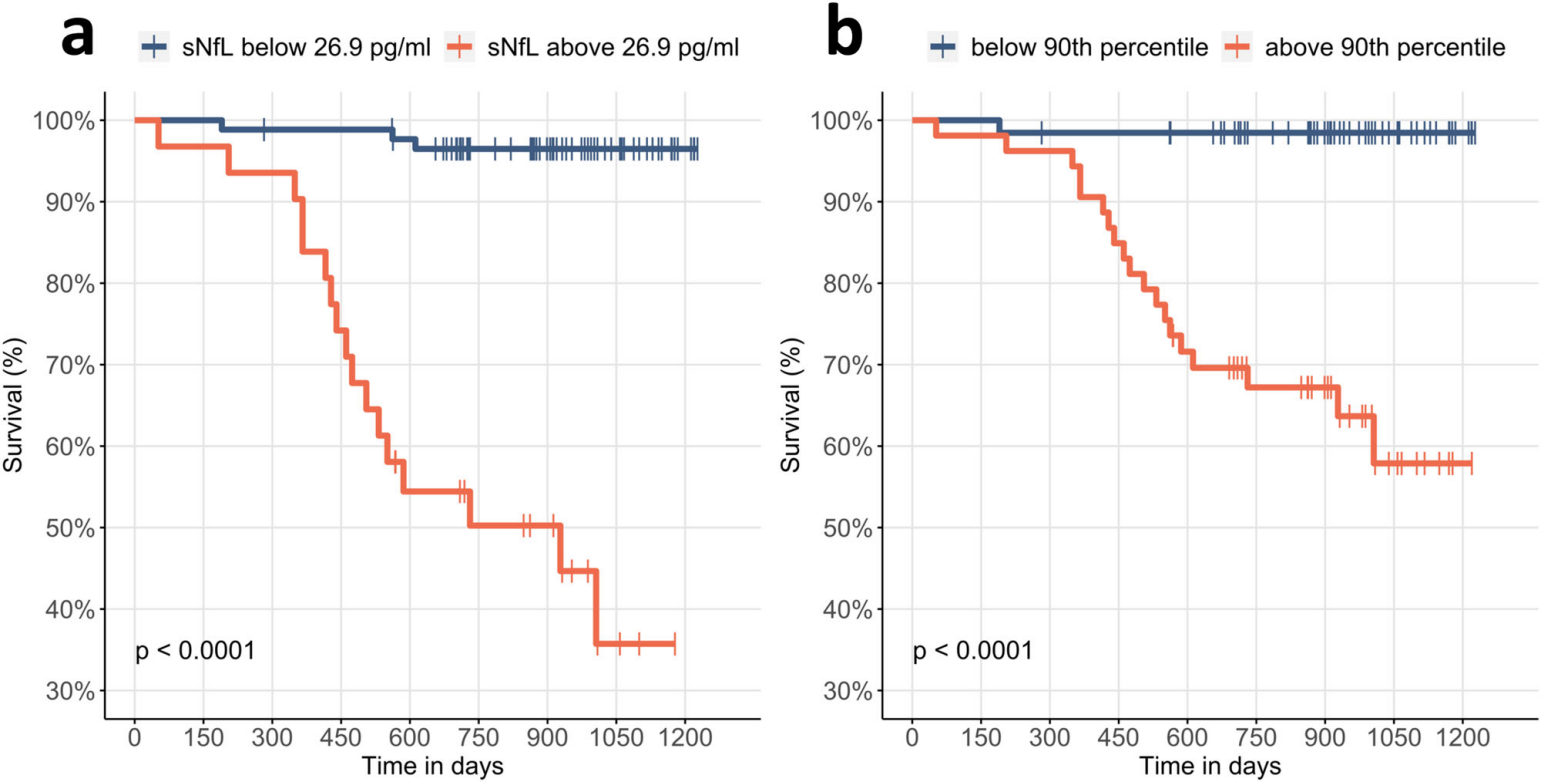
Survival curve for death during follow-up. The groups for the Kaplan–Meier estimates were determined by maximizing the Youden index for unadjusted sNfL values (**a**) and population-based age-adjusted sNfL percentiles (**b**). Both cutoff values show a highly significant difference in survival for the resulting groups (*p* < 0.001). Groups below the cutoff are depicted in orange and above the cutoff in blue.

Survival differed significantly between patients above and below this cutoff value (*p* < 0.001 Log-rank test, Fig. 3). Patients with sNfL values ≥ 26.9 pg/ml showed a median survival of only 30.6 months (5th percentile = 16.6 months; 95th percentile not in the range of follow-up). Using a median split and a quartile split as threshold values, groups similarly showed significant differences in survival (*p* < 0.001 for both, Supplementary Fig. 1).

Absolute sNfL values can differ between laboratories. In order to provide a cutoff value that can be easily adapted to other cohorts, we converted the absolute values in our cohort into age-adjusted percentiles using the Serum Neurofilament Light Chain Reference App^28^. Using the same procedure as described above, we found an AUC of 0.89 (0.83–0.96, Fig. 2b) for the prediction of death during follow-up. The optimal cutoff value based on the Youden index was 90% (Sensitivity = 95.0%, Specificity = 65.3%).

We further evaluated ROC curves for age at baseline and disease duration, the presence of milestones, MoCA score, UPDRS III score or Hoehn & Yahr stage (Table 3). Although there were no significant differences between the AUC of sNfL and MoCA score, UPDRS III score or the number of milestones (*p* = 0.443; *p* = 0.183, and *p* = 0.641, respectively), sNfL was the only predictor of death during follow-up, whose AUC was significantly higher than the combined AUC of age and disease duration (0.91 (0.85–0.97) vs 0.80 (0.69–0.91), *p* = 0.038) or the AUC of Hoehn & Yahr stage (0.92 (0.87–0.97) vs 0.82 (0.73–0.91), *p* = 0.010). ROC curves for all baseline clinical characteristics are depicted in Supplementary Fig. 2.

**Table 3:** Area under ROC curves for death during 3-year follow-up.

Characteristic	AUC	95% CI	*p* value vs sNfL	*p* value vs baseline age + disease duration
sNfL	0.91	0.85–0.97	-	**0.038**
Age-adjusted sNfL percentile	0.89	0.83–0.96	0.198	0.147
MoCA score	0.86	0.76–0.97	0.443	0.264
UPDRS III score	0.85	0.77–0.94	0.183	0.299
Number of milestones	0.90	0.81–0.98	0.641	0.112
Hoehn & Yahr stage	0.82	0.73–0.91	**0.010**	0.767
Age at baseline + disease duration	0.80	0.69–0.91	**0.038**	-

AUC under ROC curves between predictors was compared using the Delong method. *AUC* area under the curve, *CI* confidence interval, *MoCA* Montreal Cognitive Assessment, *ROC* receiver operator characteristics, *sNfL* serum neurofilament light chain, *UPDRS III* Unified Parkinson’s Disease Rating Scale part III. *p* values of significant differences with sNfL or a logistic regression model containing age and disease duration are marked in bold.

## Discussion

In the present study, the occurrence of disability milestones correlated with accelerated neurodegeneration as measured by a higher value of the blood-based biomarker sNfL. The more clinical milestones a patient had reached, the higher the levels of sNfL appeared to be. This indicates that late-stage PD, after the onset of disability milestones, is associated with accelerated neurodegeneration, and clinical deterioration not only results from functional decompensation. Furthermore, we identified sNfL as a good predictor of all-cause mortality in our advanced patient cohort.

Several well-made studies across different neurological diseases (including PD^29–33^) have shown that higher NfL values, measured in blood or cerebrospinal fluid, are consistently associated with clinical and radiological signs of neurodegeneration^21,34,35^. In PD, accordingly, NfL was shown to predict the onset of morbidity milestones and also the progression to more milestones and all-cause mortality^36^. Higher NfL is also associated with brain volume reduction, a higher number of neuronal comorbidities and all-cause mortality in the general aging population^16,37,38^. In our view, however, these studies have not fully appreciated the meaning of this association. NfL particularly does not report cumulative neuronal damage but reports the extent of currently ongoing neuronal injury as it has been demonstrated that NfL levels rise during insults that are associated with acute neuronal damage (e.g., traumatic brain injury, active multiple sclerosis or operations) and more importantly fall off again, after the initial period of damage has subsided^27,39–41^. The well-established correlation between sNfL and neurodegeneration, coupled with its temporal dynamics following acute insults, as well as its association with clinical milestones and mortality as demonstrated in this study, may therefore provide compelling evidence for an accelerated rate of neurodegeneration in advanced stages of PD.

Accelerated neurodegeneration in late-stage PD initially appears contradictory to the major dopaminergic neuron degeneration at early PD^5^. This apparent conflict can be resolved by considering the progressive spread of alpha-synuclein pathology, initially limited to the brainstem and midbrain but eventually affecting the entire brain^9,42^. While neuronal somata degeneration is most notable for dopaminergic and other catecholaminergic neurons^43^, brain-wide loss of neuronal processes and synapses likely occurs in Braak stages 5 and 6^9^. Our findings might therefore be consistent with a more widespread alpha-synuclein pathology-induced neuro-axonal damage in late-stage PD. Moreover, the strongest clinical predictor of death, cognitive decline, may result from extensive cortical damage in advanced Braak stages.

In order to exploit the predictive power of sNfL for patient counseling and trial design, we sought to determine the strongest predictors of all-cause mortality within the observation period, obtaining similar results for log(NfL), the number of clinical milestones, MoCA scores and UPDRS III scores. However, there was a trend for higher predictive accuracy using log(NfL) and it was the only predictive marker that outperformed a combined model of age at baseline, disease duration and Hoehn & Yahr stage. We then corrected for patient age since sNfL levels increase with age, and age is a major risk factor for the incidence of PD, disease progression, and death. The association of sNfL with patient survival was still observed when patient age was corrected for, either by including age in a linear model or by using age-dependent sNfL percentiles (Supplmentary Table 2 and Figs. 2, 3b). Interestingly, models that include sNfL without age correction performed somewhat better than age-corrected models, suggesting that age might play a distinct role in the increase in neurodegeneration. Since aging affects many cellular processes that predispose to neurodegeneration, the accumulation of age-related somatic damage combined with a failure of compensatory mechanisms may contribute to an acceleration of PD with age^44^. This is also consistent with the hypothesis that the increasing values of sNfL observed in an ageing population are in part caused by age-associated neurodegeneration. Accordingly, sNfL levels have been shown to increase with neuronal comorbidities and brain volume reduction^16,37^.

Interestingly, we found a higher predictive power for sNfL with respect to death during follow-up than observed in previous studies that have used a similar methodology and comparable or longer periods of follow-up (AUC for death during follow-up predicted by age-adjusted sNfL (95% CI): 0.89 (0.83–0.96) vs 0.68; HR for death during follow-up (95% CI): 13.94 (4.93–39.3) vs 1.94 (1.36–2.76)^36,45^. These discrepancies could reflect an association between the predictive ability of sNfL and disease duration or disease stage, as the study by Vijiaratnam et al. included only patients with de novo PD (disease duration: 1.3 ± 0.9 years) and patients in the study by Rödström et al. had a shorter disease duration (7.9 ± 5.1 years) compared to our cohort (15 ± 7 years)^36,45^. Furthermore, in the study by Rödström et al 15.5% of participants were excluded because they died less than 2 years after follow-up, potentially obfuscating the relationship between sNfL and death. The predictive ability of sNfL for adverse outcomes could therefore improve over the course of the disease, which could be connected to accelerated neurodegeneration in advanced disease stages.

Limitations of our study include the reliance on a single center and a single marker for neurodegeneration. In addition, this study was based on a single measurement of sNfL, which potentially has a lower predictive value compared to the longitudinal change of sNfL^21^. However, previous studies by others have shown that a single measurement of sNfL can be considered a good marker for ongoing neurodegeneration around the time of the measurement (see above). Another limitation of the study is the retrospective character of the follow-up chart review that did not allow a specification for the cause of death. Furthermore, it needs to be considered that our cohort contained a high percentage of PD patients with chronic DBS. Although we have shown in a previous study that DBS treatment does not alter the levels of sNfL in patients with PD, the results presented in this article should be fully validated in patients without DBS.

In conclusion, our findings suggest that the rate of ongoing neurodegeneration is higher in advanced PD as defined by the presence of morbidity milestones compared to earlier disease stages. A better understanding of stage-dependent neurodegeneration dynamics and potential accelerators may be helpful to further characterize the respective pathophysiological bases which in turn could help identify new therapeutic targets. Our results further suggest that disease-modifying therapies may continue to hold significant value even in the late stages of the disease and that particularly debilitating symptoms like dementia or loss of physical independence could possibly be prevented, if the neurodegenerative process is halted in that phase. Consequently, it is imperative that research and therapeutic endeavors also encompass the prevention and management of these crucial aspects. sNfL could therefore help to identify patients at risk for clinical deterioration and in addition serve as an objective outcome measure for neuroprotective substance trials.

## Methods

### Study population and design

In order to increase the probability of morbidity milestones occurring during the observation period, patients beyond the early-stage (“honeymoon”) were selected, defined for this study by a disease duration of at least 5 years^46^. The study was approved by the institutional review board of the Technische Universität Dresden (EK533122019, EK487122016) and written informed consent was obtained from all participants before inclusion in the study. Participants were recruited at the Department of Neurology of the Dresden University Hospital. The study included 88 patients on chronic deep brain stimulation (DBS) treatment, which were enrolled in a structured observational register study and 30 patients on best medical treatment, which were subjected to a solely chart-based review of clinical data. Baseline sNfL was measured during a routine clinical visit. At the same time, clinical and demographic data were collected, including the presence of clinical milestones, information about the motor and cognitive status (Hoehn & Yahr stage, UPDRS part III, Montreal cognitive assessment (MoCA)) as well as the presence of disease-related complications. Three years after the inclusion of the first patient, we performed a chart review to determine all-cause mortality and the time point of death.

### Serum NfL measurements

Serum samples were stored at −20 °C and sNfL measurements were performed as described previously, using the Advantage NF-Light Singleplex-Kit on a Simoa HD-1 instrument (Quanterix)^27,47^. Calibrators and diluted serum samples were measured in duplicates. Both the mean intraassay coefficient of variation of duplicates and the mean interassay coefficient of variation were <10%.

### Statistical analyses

To normalize the right-skewed distribution of sNfL, natural log-transformation (logNfL) was used in linear models and Cox proportional-hazards models, as described by others^19^. Age-adjusted sNfL percentiles and Z scores were calculated with the Serum Neurofilament Light Chain Reference App, which uses a reference database derived from over 5000 participants without central nervous system disorders^28^. All Cox proportional-hazards regression models were adjusted for age at baseline, disease duration and sex. The proportional-hazards assumption was tested based on visual inspection of the scaled Schoenfeld residuals and *χ*^2^ test.

Comparisons between groups were carried out using the Mann–Whitney *U* Test. Correlations were assessed with Spearman’s rank test. For multiple group comparisons, the Kruskal–Wallis test with the posthoc Dunn-test corrected for false discovery rate with the Benjamini-Hochberg method was used.

To compute cutoff values for the prediction of death, we used binary regression models and fitted receiver operator curves. The optimal sensitivity and specificity were calculated based on the Youden Index determined with the R-package cutpointr. The computed Youden Index was used to dichotomize our sample to receive Kaplan–Meier curves depending on the respective sNfL or age-adjusted sNfL percentile cutoff value. Kaplan–Meier curves were compared with log-rank tests and corrected for false discovery rate with the Benjamini–Hochberg method if necessary.

Data are depicted either as mean with standard deviation or median with 95% confidence interval if not otherwise stated. Statistical analyses were performed with R-Studio (packages: pROC, survival, survminer, cutpointr) or Python (packages: scipy, pingouin, statsmodels, kaplan–meier). The sample size was based on the size of our patient cohort and not determined by a prospective sample size estimate.

### Supplementary information

Supplemental Material

Related Manuscript File

## Data Availability

The data that support the findings of this study are available from the corresponding author upon request.

## References

[1] Heinzel S (2019). Update of the MDS research criteria for prodromal Parkinson’s disease. Mov. Disord..

[2] Pagano G (2022). Trial of prasinezumab in early-stage Parkinson’s disease. N. Engl. J. Med..

[3] Antonini A (2018). Developing consensus among movement disorder specialists on clinical indicators for identification and management of advanced Parkinson’s disease: a multi-country Delphi-panel approach. Curr. Med. Res. Opin..

[4] Coelho M, Ferreira JJ (2012). Late-stage Parkinson disease. Nat. Rev. Neurol..

[5] Kordower JH (2013). Disease duration and the integrity of the nigrostriatal system in Parkinson’s disease. Brain.

[6] Barone P (2010). Neurotransmission in Parkinson’s disease: beyond dopamine. Eur. J. Neurol..

[7] Titova N, Padmakumar C, Lewis SJG, Chaudhuri KR (2017). Parkinson’s: a syndrome rather than a disease?. J. Neural Transm..

[8] Karachi C (2010). Cholinergic mesencephalic neurons are involved in gait and postural disorders in Parkinson disease. J. Clin. Invest.

[9] Braak H (2003). Staging of brain pathology related to sporadic Parkinson’s disease. Neurobiol. Aging.

[10] Halliday G, Hely M, Reid W, Morris J (2008). The progression of pathology in longitudinally followed patients with Parkinson’s disease. Acta Neuropathol.

[11] Shen W, Zhai S, Surmeier DJ (2022). Striatal synaptic adaptations in Parkinson’s disease. Neurobiol. Dis..

[12] Falkenburger B, Kalliakoudas T, Reichmann H (2022). Adaptive changes in striatal projection neurons explain the long duration response and the emergence of dyskinesias in patients with Parkinson’s disease. J. Neural Transm.

[13] Kempster PA, O’Sullivan SS, Holton JL, Revesz T, Lees AJ (2010). Relationships between age and late progression of Parkinson’s disease: a clinico-pathological study. Brain.

[14] Schnalke N (2023). Morbidity milestones demonstrate long disability-free survival in Parkinson’s disease patients with deep brain stimulation of the subthalamic nucleus. Mov. Disord. Clin. Pract.

[15] Barro C, Chitnis T, Weiner HL (2020). Blood neurofilament light: a critical review of its application to neurologic disease. Ann. Clin. Transl. Neurol..

[16] Khalil M (2020). Serum neurofilament light levels in normal aging and their association with morphologic brain changes. Nat. Commun..

[17] Bäckström D (2020). NfL as a biomarker for neurodegeneration and survival in Parkinson disease. Neurology.

[18] Marques TM (2019). Serum NFL discriminates Parkinson disease from atypical parkinsonisms. Neurology.

[19] Mollenhauer B (2020). Validation of serum neurofilament light chain as a biomarker of Parkinson’s disease progression. Mov. Disord..

[20] Elmers J, Colzato LS, Akgün K, Ziemssen T, Beste C (2023). Neurofilaments—small proteins of physiological significance and predictive power for future neurodegeneration and cognitive decline across the life span. Ageing Res. Rev..

[21] Preische O (2019). Serum neurofilament dynamics predicts neurodegeneration and clinical progression in presymptomatic Alzheimer’s disease. Nat. Med.

[22] Khalil M (2018). Neurofilaments as biomarkers in neurological disorders. Nat. Rev. Neurol..

[23] Delcoigne B (2020). Blood neurofilament light levels segregate treatment effects in multiple sclerosis. Neurology.

[24] Olsson B (2019). NFL is a marker of treatment response in children with SMA treated with nusinersen. J. Neurol..

[25] Miller TM (2022). Trial of antisense oligonucleotide tofersen for SOD1 ALS. N. Engl. J. Med..

[26] Hansson O. Biomarkers for neurodegenerative diseases. *Nat Med*. **27**, 954–963 (2021). 10.1038/s41591-021-01382-x 34083813

[27] Frank A (2022). Serum neurofilament indicates that DBS surgery can cause neuronal damage whereas stimulation itself does not. Sci. Rep..

[28] Benkert P (2022). Serum neurofilament light chain for individual prognostication of disease activity in people with multiple sclerosis: a retrospective modelling and validation study. Lancet Neurol..

[29] Lin CH (2019). Blood NfL: a biomarker for disease severity and progression in Parkinson disease. Neurology.

[30] Niemann L (2021). Serum neurofilament is associated with motor function, cognitive decline and subclinical cardiac damage in advanced Parkinson’s disease (MARK-PD). Park Relat. Disord..

[31] Ye R (2021). Serum NFL levels predict progression of motor impairment and reduction in putamen dopamine transporter binding ratios in de novo Parkinson’s disease: an 8-year longitudinal study. Park Relat. Disord..

[32] Aamodt, W. W. et al. Neurofilament light chain as a biomarker for cognitive decline in parkinson disease. *Mov. Disord.* **36**, 2945–2950 (2021). 10.1002/mds.28779 PMC8688198 34480363

[33] Buhmann C (2022). Age-adjusted serum neurofilament predicts cognitive decline in Parkinson’s Disease (MARK-PD). Mov. Disord..

[34] Chelban V (2022). Neurofilament light levels predict clinical progression and death in multiple system atrophy. Brain.

[35] Cantó E (2019). Association between serum neurofilament light chain levels and long-term disease course among patients with multiple sclerosis followed up for 12 years. JAMA Neurol..

[36] Ygland Rödström E, Mattsson-Carlgren N, Janelidze S, Hansson O, Puschmann A (2022). Serum neurofilament light chain as a marker of progression in parkinson’s disease: long-term observation and implications of clinical subtypes. J. Parkinsons Dis..

[37] Ladang, A. et al. Neurofilament light chain concentration in an aging population. *Aging Clin. Exp. Res*. **34**, 331–339 (2022). 10.1007/s40520-021-02054-z PMC8847291 35018623

[38] Nguyen AD (2022). Serum neurofilament light levels are predictive of all-cause mortality in late middle-aged individuals. eBioMedicine.

[39] Shahim P, Zetterberg H, Tegner Y, Blennow K (2017). Serum neurofilament light as a biomarker for mild traumatic brain injury in contact sports. Neurology.

[40] Kuhle J (2019). Blood neurofilament light chain as a biomarker of MS disease activity and treatment response. Neurology.

[41] Bergman J (2016). Neurofilament light in CSF and serum is a sensitive marker for axonal white matter injury in MS. Neurol. Neuroimmunol. NeuroInflamm..

[42] Halliday G, Lees A, Stern M (2011). Milestones in Parkinson’s disease-clinical and pathologic features. Mov. Disord..

[43] Surmeier DJ, Obeso JA, Halliday GM (2017). Selective neuronal vulnerability in Parkinson disease. Nat. Rev. Neurosci..

[44] Hindle JV (2010). Ageing, neurodegeneration and Parkinson’s disease. Age Ageing.

[45] Vijiaratnam N (2022). Combining biomarkers for prognostic modelling of Parkinson’s disease. J. Neurol. Neurosurg. Psychiatry.

[46] Stocchi F, Jenner P, Obeso JA (2010). When do levodopa motor fluctuations first appear in Parkinson’s disease?. Eur. Neurol..

[47] Ziemssen T, Akgün K, Brück W (2019). Molecular biomarkers in multiple sclerosis. J. Neuroinflamm..

