# Supplementary material for: Serum neurofilament indicates accelerated neurodegeneration and predicts mortality in late-stage Parkinson’s disease
Source: NPJ Parkinsons Dis. 2024 Jan 9;10:14. doi: 10.1038/s41531-023-00605-x (PMC10776839; doi:10.1038/s41531-023-00605-x)
Supplement: Supplementary file 1 — Supplemental Material [file 41531_2023_605_MOESM1_ESM.pdf]

# Supplementary Data

**SUPPLEMENTARY TABLE 1. Comparison between groups for baseline characteristics.**

| | **No milestone vs ≥ 1 milestone at baseline** | **Alive vs deceased during follow-up** |
|---|---|---|
| **Age at baseline** | 0.001 | < 0.001 |
| **Age at onset** | 0.265 | 0.585 |
| **Disease duration** | < 0.001 | < 0.001 |
| **Sex** | 0.866 | 1.0 |
| **Hoehn & Yahr stage** | < 0.001 | < 0.001 |
| **LEDD** | 0.361 | 0.606 |
| **UPDRS III score (on medication)** | 0.010 | <0.001 |
| **MOCA score** | < 0.001 | < 0.001 |
| **Total number of milestones at baseline** | < 0.001 | < 0.001 |
| **Serum NFL** | < 0.001 | < 0.001 |

*A Mann-Whitney-U-Test was used for all comparisons between groups except for the categorical variable Sex (Chi-Square-Test used instead).*

**SUPPLEMENTARY TABLE 2. Univariate linear regression models.**

| | **logNfL** | | **Population-based age-adjusted sNfL percentile** | |
|---|---|---|---|---|
| | **$R^2$** | **p-value** | **$R^2$** | **p-value** |
| **Age at baseline** | 0.29 | < 0.001 | 0 | 0.395 |
| **Disease duration** | 0.21 | < 0.001 | 0.06 | 0.004 |
| **Hoehn & Yahr stage** | 0.23 | < 0.001 | 0.07 | 0.002 |
| **UPDRS III score (on medication)** | 0.18 | < 0.001 | 0.01 | 0.130 |
| **MoCA score** | 0.27 | < 0.001 | 0.07 | 0.004 |
| **Total number of milestones at baseline** | 0.51 | < 0.001 | 0.24 | < 0.001 |
| **Milestone dementia** | 0.34 | < 0.001 | 0.14 | < 0.001 |
| **Milestone visual hallucinations** | 0.12 | < 0.001 | 0.08 | 0.001 |
| **Milestone recurrent falls** | 0.29 | < 0.001 | 0.18 | < 0.001 |
| **Milestone nursing home admission** | 0.27 | < 0.001 | 0.07 | 0.003 |

*The table shows $R^2$-values and p-values for univariate linear regression models with logNfL or population-based age-adjusted sNfL percentiles as dependent variable. Independent variables of each model are listed in the first column.*

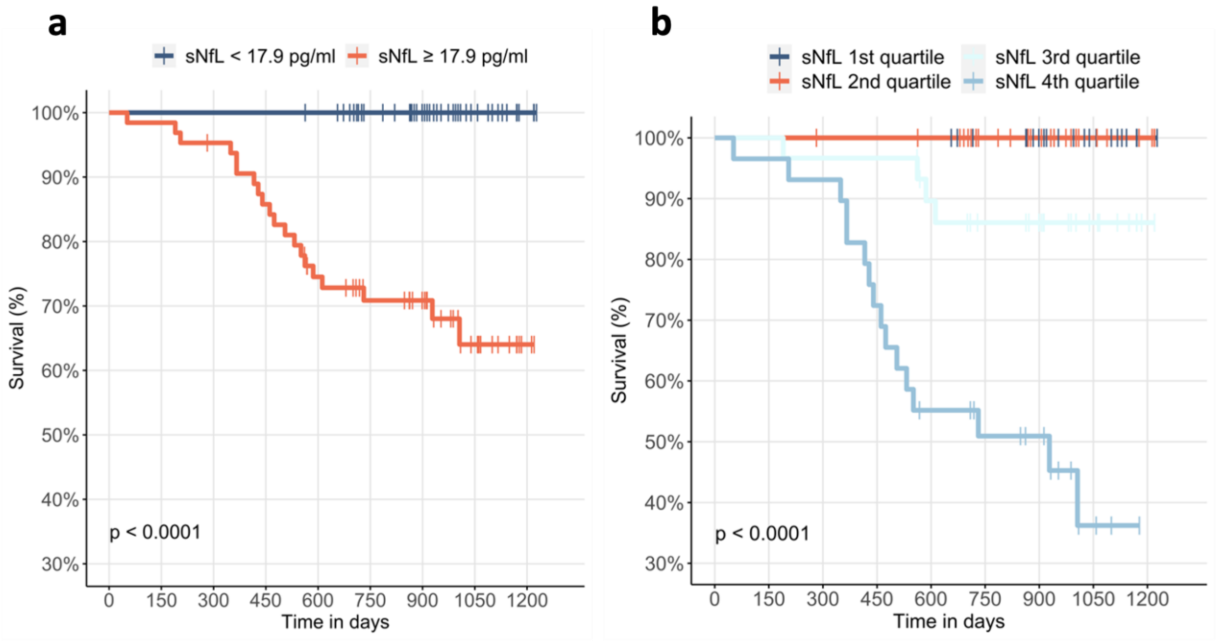


**SUPPLEMENTARY FIGURE 1. Survival curves for death during follow-up with different sNfL cut-off values.** *Median split on sNfL (**a**) or a quartile-based split on sNfL (**b**).*

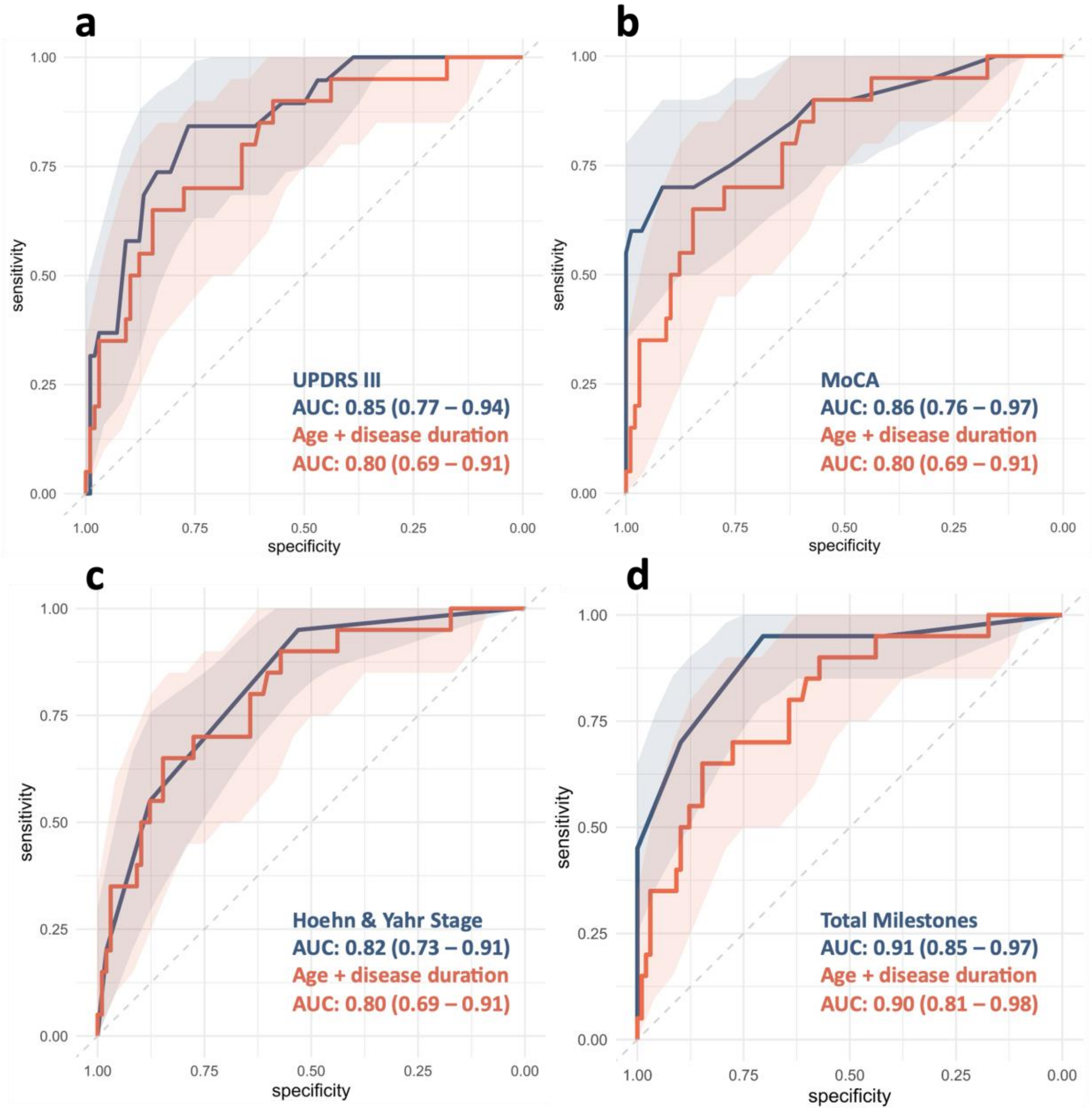


**SUPPLEMENTARY FIGURE 2. ROC curves for the prediction of death during follow-up.** ROC curves of the baseline variables UPDRS III score (**a**), MoCA score (**b**), Hoehn & Yahr stage (**c**) and the number of milestones at baseline (**d**) are depicted (blue curves). *For reference, a ROC curve of a logistic regression model containing age and disease duration was added in all panels (orange curves). 95 % confidence intervals (bootstrap method, 2000 stratified intervals) are depicted around each ROC curve. Abbreviations: AUC – area under the curve; MoCA – Montreal Cognitive Assessment; UPDRS III – Unified Parkinson's Disease Rating Scale Part III.*
